# Recent advances in chemotherapy for pancreatic cancer: evidence from Japan and recommendations in guidelines

**DOI:** 10.1007/s00535-020-01666-y

**Published:** 2020-01-29

**Authors:** Takuji Okusaka, Junji Furuse

**Affiliations:** 1 Department of Hepatobiliary and Pancreatic Oncology, National Cancer Center Hospital, 5-1-1 Tsukiji, Chuo-ku, Tokyo, 104-0045 Japan; 2 Department of Medical Oncology, Faculty of Medicine, Kyorin University, Tokyo, Japan

**Keywords:** Pancreatic ductal adenocarcinoma, Adjuvant therapy, Immunotherapy, Targeted therapy, Actionable mutation

## Abstract

The prognosis of patients with pancreatic cancer continues to remain dismal, even though numerous trials have been conducted to establish more effective therapies in Japan and throughout the world. Recent advances in treatment have been characterized by the use of novel combinations of conventional cytotoxic chemotherapies. Especially in Japan, S-1 has become one of the most widely used cytotoxic agents for the treatment of pancreatic cancer, after clinical evidence was established of the survival benefit offered by this drug for patients with resectable or unresectable pancreatic cancer. Unfortunately, with the exception of erlotinib, no targeted treatment strategies have been approved for pancreatic cancer. However, following an increase in interest in drug development in recent years, proactive attempts have been made to develop new therapeutic strategies, including neoadjuvant chemotherapy for patients with resectable or borderline resectable pancreatic cancer, multi-agent combination chemotherapy for patients with advanced pancreatic cancer, and therapies with new targeted agents or immuno-oncologic agents for patients with pancreatic cancer bearing specific gene mutations.

## Introduction

In Japan, along with the rapidly aging population, the number of patients with pancreatic cancer is also rapidly increasing, similar to the case for lung and colorectal cancer [1]. The number of deaths from pancreatic cancer in Japan has recently exceeded that from liver cancer, and pancreatic cancer now ranks as the fourth leading cause of death from cancer. In the majority of patients with pancreatic cancer, the cancer is already at an advanced unresectable stage at the time of diagnosis. Even in patients with resectable tumor at diagnosis who are treated by surgery, recurrence often occurs in the early phase after the operation. Thus, the prognosis of pancreatic cancer remains extremely poor [2].

The two most important approaches to improve the prognosis of pancreatic cancer are (1) to establish a better diagnostic method that would enable detection of this cancer at a resectable stage, and (2) to establish effective non-surgical treatments for patients with recurrent or unresectable pancreatic cancer. Until recently, there were a few effective non-surgical treatments. However, numerous clinical studies have been conducted in recent years, which have led to the establishment of new standard treatments that offer survival advantage. Several phase III studies have also been conducted in Japan, which have led to the establishment of standard treatments unique to pancreatic cancer patients in Japan.

**Fig. 1:**
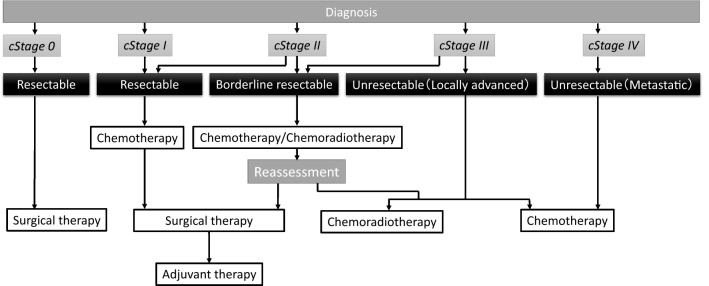
Algorithm for the treatment of pancreatic cancer according to the Clinical Practice Guidelines for Pancreatic Cancer 2019 from the Japan Pancreas Society. The clinical cancer stage (cStage) classification and resectability classification are based on the General Rules for the Study of Pancreatic Cancer, Seventh Edition, The Japan Pancreas Society

**Fig. 2:**
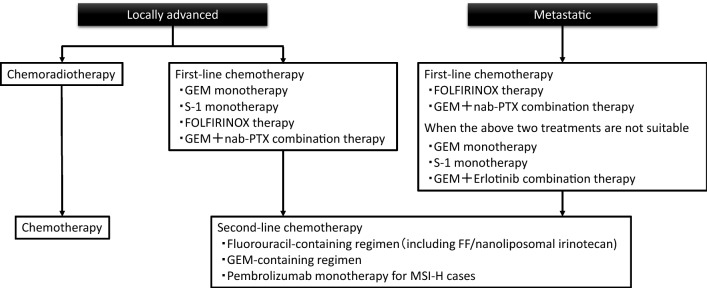
Algorithm for chemotherapy of pancreatic cancer according to the Clinical Practice Guidelines for Pancreatic Cancer 2019 published by the Japan Pancreas Society. *GEM* gemcitabine, nab-*PTX* nab-paclitaxel, *FF* fluorouracil + calcium folinate

In this article, we review the recent developments in chemotherapy for pancreatic cancer, including those based on evidence from clinical trials conducted in Japan, the current standard therapies recommended in guidelines in Japan (Figs. 1 and 2) and overseas, and global attempts to establish new strategies to overcome this disease with a grim prognosis.

## Neoadjuvant chemotherapy

Patients with pancreatic cancer are known to show high recurrence rates even after curative resection, and the prognosis of patients with recurrent disease is extremely poor. To prevent or delay the development of recurrence after resection and to improve the prognosis in patients with resectable tumor, numerous clinical trials of adjuvant therapy, including chemotherapy and chemoradiotherapy, administered before and/or after resection, have been actively undertaken both in Japan and overseas. Among the several types of adjuvant therapy, postoperative adjuvant chemotherapy has come to be recognized as a standard treatment strategy globally, based on demonstration in recent phase III studies of its ability to improve the long-term prognosis of pancreatic cancer patients. On the other hand, until recently, no solid evidence from large-scale randomized-controlled studies had been established the survival benefit of neoadjuvant (preoperative) therapy. In 2018 to 2019, one phase III study each of neoadjuvant therapy was conducted in Japan and overseas (Table 1).

**Table 1:** Major randomized phase III trials of neoadjuvant treatments with reported results for pancreatic cancer

Study	Treatments	No. of patients	Median disease-free survival (months)	*p* value	Median survival (months)	*p* value
Prep-02/JSAP-05 2019	Gemcitabine/S-1	182	14.28	0.028	36.72	0.015
	Up-front surgery	180	11.28		26.65	
PREOPANC-1 2018	Gemcitabine/radiation	119	9.9	0.023	17.1	0.074
	Up-front surgery	127	7.9		13.7	

*Prep* Study group of preoperative therapy for pancreatic cancer, *JSAP* Japanese Study Group of Adjuvant Therapy for Pancreatic cancer, *PREOPANC* Preoperative radiochemotherapy versus immediate surgery for resectable and borderline resectable pancreatic cancer

The results of the phase III study (Prep-02/JSAP-05 Study) of neoadjuvant chemotherapy with gemcitabine plus S-1 for pancreatic cancer patients scheduled for resection conducted in Japan were reported at the American Society of Clinical Oncology-—Gastrointestinal Cancers Symposium (ASCO-GI) 2019; the study showed that the overall survival (OS) was significantly better in the neoadjuvant therapy group as compared to that in the upfront surgery group [hazard ratio (HR) 0.72, *p* = 0.015] [3–5]. Approximately 80% of patients enrolled in this study had resectable pancreatic cancer at diagnosis, although the study also included patients with borderline resectable pancreatic cancer with portal vein invasion. A subgroup analysis showed better treatment outcomes in patients with resectable tumor and a trend towards better survival in patients with borderline resectable cancer assigned to the neoadjuvant therapy arm [5].

The overseas phase III study (PREOPANC-1 study) to confirm the survival benefit of neoadjuvant chemoradiotherapy was conducted in The Netherlands in patients with resectable and borderline resectable pancreatic cancer [6]. The results, reported at ASCO 2018, showed a trend towards a better OS in the neoadjuvant therapy arm as compared to the immediate surgery arm, although the difference was not statistically significant (HR 0.74, *p* = 0.074).

Prior to these two studies mentioned above, no phase III study had demonstrated the benefits of neoadjuvant therapy for patients with resectable pancreatic cancer [7, 8]; therefore, Japanese guidelines had not recommended neoadjuvant therapy for patients with pancreatic cancer until recently [9, 10]; the same is true of guidelines in other countries overseas, which still do not recommend neoadjuvant therapy as standard treatment for patients with resectable pancreatic cancer with exceptions for those with high-risk factors [11–14]. Based on the results of the Prep-02/JSAP-05 study, however, the latest Japanese guidelines (Clinical Practice Guidelines for Pancreatic Cancer 2019) recommend gemcitabine plus S-1 combination therapy (GS therapy) as a standard neoadjuvant therapy for patients with resectable pancreatic cancer [15, 16]. Since the Prep-02/JSAP-05 study was conducted only in Japan and use of S-1 is not as feasible in Western populations, as mentioned later, until now, GS therapy is recognized as a standard therapy only in Japan and China [13, 17, 18].

For patients with borderline resectable pancreatic cancer, Japanese guidelines recommend neoadjuvant therapy, in general, but have refrained from recommending any specific regimens [15, 16]. Although guidelines in many other countries also recommend neoadjuvant therapy for borderline resectable pancreatic cancer, no consensus on any standard regimens has been established in any country until date [11–14]. Among the several ongoing randomized-controlled trials of treatments for borderline resectable pancreatic cancer (Table 2) [19–27], a phase II/III study of neoadjuvant therapy with gemcitabine plus nab-paclitaxel therapy versus chemoradiotherapy with S-1 is under way in Japan; this study is expected to provide specific new evidence for the establishment of a standard regimen for borderline resectable pancreatic cancer [21].

**Table 2:** Major ongoing randomized trials of neoadjuvant treatments for borderline resectable pancreatic cancer

Study	Treatments	Eligibility	Phase	No. of patients	Primary endpoint	Study Start	Estimated Study Completion	Country
UVA-PC-PD101 NCT02305186	Radiation/capecitabine/pembrolizumab	R BR	Phase 1/2	56	Number of tumor-infiltrating lymphocytes (TILs)	Mar 2015	Dec 2020	US
	Radiation/capecitabine							
NCT02717091	FOLFIRINOX	BR	Phase 2	50	R0 resection rate	Jul 2015	Jun 2020	Japan
	gemcitabine/nab-paclitaxel							
GABARNANCE Trial UMIN000026858	S1 + radiation	BR	Phase 2/3	110	Overall survival	Apr 2017	Sep 2022	Japan
	Gemcitabine/nab-paclitaxel							
PANDAS-PRODIGE 44 NCT02676349	mFOLFIRINOX + radiation/capecitabine	BR	Phase 2	90	R0 resection rate	Oct 2016	Jan 2026	France
	mFOLFIRINOX							
Alliance Trial A021501 NCT02839343	mFOLFIRINOX + radiation	BR	Phase 2	126	18 months overall survival rate	Dec 2016	Mar 2020	Canada, US
	mFOLFIRINOX							
BRPCNCC-1 NCT03777462	Gemcitabine/nab-paclitaxel	BR	Phase 2	150	Overall survival	Apr 2019	Dec 2021	China
	Gemcitabine/nab-paclitaxel + radiation							
	S1/Nab-paclitaxel + radiation							
NCT01458717	radiation/gemcitabine	BR	Phase 2/3	58	2-year survival rate	Nov 2011	Jan 2018	Korea
	Upfront surgery							
NEOLAP NCT02125136	Gemcitabine/nab-paclitaxel	BR LA	Phase 2	168	Conversion rate	Nov 2014	Oct 2020	US
	Gemcitabine/nab-paclitaxel + mFOLFIRINOX							
NCT03983057	mFOLFIRINOX/Anti-PD-1 antibody	BR LA	Phase 3	830	Progression-free survival	Apr 2019	Apr 2021	China
	mFOLFIRINOX							

R resectable, *BR* borderline resectable, *LA* locally advanced, *mFOLFIRINOX* modified-FOLFIRINOX

## Adjuvant chemotherapy

Randomized-controlled trials comparing postoperative adjuvant chemotherapy and resection alone have been conducted since the 1990s, mainly in Europe and Japan (Table 3). In the CONKO-001 trial conducted in Germany and Austria, 354 patients who had undergone resection for pancreatic cancer were randomly assigned to receive postoperative adjuvant chemotherapy with gemcitabine alone or resection alone [28, 29]. The results showed a significantly prolonged recurrence-free survival in the adjuvant chemotherapy arm. While no significant prolongation of the OS was noted initially (*p* = 0.06) [28], a subsequent analysis performed after long-term follow-up revealed significant prolongation of not only the recurrence-free survival, but also of the OS [29]. In the JSAP-02 study conducted in Japan, 118 patients who had undergone resection for pancreatic cancer were randomly assigned to receive postoperative adjuvant chemotherapy with gemcitabine alone or resection alone [30]. Consistent with the initial results of the CONKO-001 trial, significant prolongation of the recurrence-free survival was observed in the gemcitabine-alone arm. The European Study Group of Pancreatic Cancer (ESPAC) conducted the ESPAC-3 Study in Europe, Australia, Japan, and Canada, in which 1088 patients who had undergone resection for pancreatic cancer were randomly assigned to receive postoperative adjuvant chemotherapy with either fluorouracil plus folinate calcium or gemcitabine alone [31]. Although there was no significant difference in the OS between the two groups, the incidence of serious adverse events was significantly lower in the gemcitabine-alone arm than in the fluorouracil plus folinate calcium arm. These results indicate that patients receiving postoperative adjuvant chemotherapy with gemcitabine show significantly better survival outcomes than those undergoing resection alone; in addition, since serious adverse events were also less frequent in the gemcitabine arm than in the fluorouracil plus folinate calcium arm, gemcitabine could be regarded as the global standard treatment agent for postoperative adjuvant chemotherapy.

**Table 3:** Pivotal phase III trials of adjuvant treatments for pancreatic cancer

Study	Regimens	No. of patients	Median disease-free survival (months)	*p* value	Median survival (months)	*p* value
ESPAC-1 2004	Chemoradiotherapy	73	NR	NR	13.9	*p* = 0.009* *p* = 0.05^+^
	5-FU/folinic acid	75	NR		21.6	
	Chemoradiotherapy + 5-FU/folinic acid	72	NR		19.9	
	Observation	69	NR		16.9	
CONKO-001 2007	Gemcitabine	179	13.4	0.001	22.1	0.06
	Observation	175	6.9		20.2	
ESPAC-3 2010	Gemcitabine	537	14.3	0.53	23.6	0.39
	5-FU/folinic acid	551	14.1		23.0	
JASPAC-01 2016	S-1	192	22.9	0.0001	46.5	0.0001
	Gemcitabine	193	11.3		25.5	
ESPAC-4 2017	Gemcitabine plus capecitabine	364	13.9	0.082	28.0	0.032
	Gemcitabine	366	13.1		25.5	
CONKO-005 2017	Gemcitabine plus erlotinib	219	11.4	0.26	24.5	0.61
	Gemcitabine	215	11.4		26.5	
Unicancer GI PRODIGE 24/CCTG PA.6 2018	Modified FOLFIRINOX	247	21.6	0.001	54.4	0.003
	Gemcitabine	246	12.8		35.0	
APACT 2019	Gemcitabine plus nab-paclitaxel	432	19.4	0.182	40.5	0.045
	Gemcitabine	434	18.8		36.2	

*ESPAC* European Study Group for Pancreatic Cancer 1, *CONKO* Charité Onkologie, *JASPAC* Japan Adjuvant Study Group of Pancreatic Cancer, GI gastrointestinal, *PRODIGE* partenariat de recherche en oncologie digestive, *CTG* PA Clinical Trials Group Pancreatic Adenocarcinoma, *APACT* adjuvant therapy for patients with resected pancreatic cancer

^*^Chemotherapy vs. no chemotherapy

^+^Chemoradiotherapy vs. no chemoradiotherapy

In Japan, the Japan Adjuvant Study Group of Pancreatic Center (JASPAC) conducted a phase III comparative study (JASPAC 01) of postoperative adjuvant chemotherapy with gemcitabine alone versus S-1 alone in patients who had undergone resection for pancreatic cancer [32]. A total of 385 patients were enrolled, and the 5-year survival rate and median survival time were 44.1% and 46.5 months, respectively, in the S-1 group, and 24.4% and 25.5 months, respectively, in the gemcitabine group. The results demonstrated that postoperative adjuvant therapy with S-1 as compared to that with gemcitabine was associated with a significantly improved OS after resection of pancreatic cancer (HR 0.57,* p* < 0.0001). The ESPAC conducted the ESPAC-4 study, in which 730 patients who had undergone resection for pancreatic cancer were randomly assigned to receive either gemcitabine alone or combined gemcitabine plus capecitabine therapy in England, Scotland, Wales, Germany, France, and Sweden [33]. The median survival time was 25.5 months in the gemcitabine monotherapy arm and 28.0 months in the gemcitabine plus capecitabine arm. The results demonstrated that the combined gemcitabine plus capecitabine regimen yielded a significantly prolonged OS after pancreatic cancer resection as compared to gemcitabine monotherapy (HR: 0.82, *p* = 0.032). The results of the PRODIGE 24-ACCORD 24/CCTG PA 6 study, conducted in France and Canada, have also been reported; in this study, the modified FOLFIRINOX regimen was compared with gemcitabine alone as adjuvant therapy [34]. A total of 493 patients were enrolled, and the median disease-free survival, which was the primary endpoint, was 21.6 months in the modified FOLFIRINOX arm and 12.8 months in the gemcitabine monotherapy arm, demonstrating superior outcomes in the modified FOLFIRINOX arm (HR 0.58, *p* < 0.0001). In terms of the OS also, better results were obtained in the modified FOLFIRINOX arm (the median survival time was 54.4 months in the modified FOLFIRINOX arm and 35.0 months in the gemcitabine monotherapy arm; HR 0.64, *p* = 0.003). Since no clinical study has been conducted to compare S-1 alone with the combined gemcitabine plus capecitabine regimen and modified FOLFIRINOX regimen, and it is still not clear as to which of the three above regimes might be the optimal one for adjuvant therapy. In Japan and China, S-1 is frequently used as the standard treatment agent [13, 15, 16] because of its higher efficacy as compared to gemcitabine monotherapy (as suggested by the superior HR of 0.57) [32], its milder adverse effects in Asians, and its availability as an oral formulation, which can be expected to reduce the burden on the patients. On the other hand, S-1 has not been tested as adjuvant therapy in Western populations [12, 17]. Therefore, in countries including Europe and the US, the gemcitabine plus capecitabine regimen or modified FOLFIRINOX regimen is preferred and regarded as the standard [11, 12, 14]. Thus, this is another difference in the treatment practice for pancreatic cancer between Asian and Western countries.

The global phase III APACT trial evaluated adjuvant treatment with nab-paclitaxel plus gemcitabine versus gemcitabine alone in patients with resected pancreatic cancer [35]. Results of the study were reported at ASCO 2019. The primary endpoint, disease-free survival by independent review, was not met. The median disease-free survival was 19.4 months with nab-paclitaxel plus gemcitabine versus 18.8 months with gemcitabine monotherapy (HR = 0.88; *p* = 0.1824). However, the prespecified sensitivity analysis of investigator-assessed disease-free survival and interim OS were improved with nab-paclitaxel plus gemcitabine versus gemcitabine alone (HR 0.82 for both). Additional OS follow-up may better support nab-paclitaxel plus gemcitabine as an option in the adjuvant setting.

### Chemotherapy for locally advanced pancreatic cancer

Locally advanced pancreatic cancer, defined as locally invasive pancreatic cancer without obvious distant metastases, is difficult to resect because of invasion of the major arteries. Both in Japan and other countries, guidelines recommend chemoradiotherapy or chemotherapy alone for locally advanced pancreatic cancer, although there is no consensus yet on which of the two might be preferable [11–16].

In regard to chemoradiotherapy for locally advanced pancreatic cancer, in western countries, induction chemotherapy is undertaken prior to chemoradiotherapy, and is recommended by guidelines as the standard treatment option [11, 12]. The aims of induction chemotherapy are to select patients who are more likely to benefit from chemoradiotherapy and to prevent distant metastases. However, there have been no randomized-controlled trials examining the clinical benefits of induction chemotherapy, except for the JCOG1106 trial, which was a randomized phase II trial conducted in Japan [36]. In this study, the median survival time and 2-year OS in the induction chemotherapy arm receiving gemcitabine monotherapy for 12 weeks before the start of radiotherapy combined with S-1 were 17.2 months and 18.9%, respectively; no statistically significant differences were observed as compared to the corresponding values (19.0 months and 36.9%) in the group that did not receive induction chemotherapy, although a trend towards poorer outcomes was observed in the induction chemotherapy arm. There was no significant difference in the incidence of adverse reactions observed either between the two groups. This study, which is the only trial of induction chemotherapy conducted to date, failed to demonstrate any clinical benefits of induction chemotherapy. Induction chemotherapy is not a common practice in Japan. Therefore, Japanese guidelines do not recommend induction chemotherapy for patients with locally advanced pancreatic cancer [15, 16].

Historically, in clinical trials of systemic chemotherapy for advanced pancreatic cancer, both patients with locally advanced disease and patients with distant metastases have been enrolled under the umbrella term, “unresectable pancreatic cancer,” and treatments that were found to be of survival benefit in these studies have been regarded as the standard treatments for both categories of patients. However, in recent years, these two categories of patients have been classified into separate group in trials, and many phase III studies for systemic chemotherapy are now being conducted in only patients with distant metastases (Table 4). On the other hand, no definitive conclusions have been reached yet as to the standard therapy for this population; chemotherapies demonstrated to show survival benefit in patients with distant metastases are also considered highly likely to be effective in patients with locally advanced pancreatic cancer. FOLFIRINOX and the combined gemcitabine plus nab-paclitaxel regimen have been shown to prolong the survival, as compared to gemcitabine monotherapy, in patients with distant metastases [37, 38], and are, therefore, also the most highly recommended regimens in both Japanese [15, 16] and overseas guidelines [11–13] for locally advanced pancreatic cancer patients with a good performance status (PS) except British guidelines [14]. In Japan, a randomized phase II study (JCOG1407) is under way to compare the efficacy and safety of the modified FOLFIRINOX regimen and combined gemcitabine plus nab-paclitaxel regimen for patients with locally advanced pancreatic cancer, to determine the most promising chemotherapy regimen for this stage of disease [39]. This study was the world’s first randomized-controlled study comparing the two regimens, and a subsequent phase III study is being planned to compare the chemotherapy regimen that is suggested to be promising by this phase II study with chemoradiotherapy, which is also a standard treatment strategy for locally advanced pancreatic cancer. Therefore, this study is expected to contribute greatly to the establishment of evidence-based standard treatment for pancreatic cancer patients with locally advanced disease.

**Table 4:** Pivotal phase III trials evaluating first-line treatment for advanced pancreatic cancer

Study	Regimens	Eligibility	No. of patients	Response rate (%)	Median progression-free survival (months)	*p* value	Median survival (months)	Hazard ratio	*p* value
Gemcitabine vs. 5-FU 1997	Gemcitabine	LA M	63	5.4	9 weeks	0.0002	5.65	NR	0.0025
	5-FU		63	0	4 weeks		4.41		
NCIC CTG PA.3 2007	Gemcitabine plus erlotinib	LA M	285	8.6	3.75	0.004	6.24	0.82	0.038
	Gemcitabine		284	8.0	3.55		5.9		
GEST 2013	Gemcitabine plus S-1	LA M	275	29.3	5.7	< 0.001*	10.1	0.88	0.15*
	S-1		280	21.0	3.8	0.02^+^	9.7	1.0	0.001^+^
	Gemcitabine		277	13.3	4.1		8.8		
PRODIGE 4/ ACCORD 11 2011	FOLFIRINOX	M	171	31.8	6.4	< 0.001	11.1	0.57	< 0.001
	Gemcitabine		171	11.3	3.3		6.8		
MPACT 2019	Gemcitabine plus nab-paclitaxel	M	431	23	5.5	< 0.001	8.5	0.7	< 0.001
	Gemcitabine		430	7	3.7		6.7		

*L* locally advanced, *M* metastatic, *NCIC CTG PA* National Cancer Institute of Canada—Clinical Trials Group Pancreatic Adenocarcinoma, *GEST* gemcitabine and TS-1 Trial, *PRODIGE*: partenariat de recherche en oncologie digestive, *ACCORD* actions concertées dans les cancers colorectaux et digestif, *MPACT* Metastatic Pancreatic Adenocarcinoma Clinical Trial

^*^Superiority to gemcitabine

^+^Non-inferiority to gemcitabine

## Chemotherapy for metastatic pancreatic cancer

The Japanese Clinical Practice Guidelines for Pancreatic Cancer 2019 recommends FOLFIRINOX therapy or combined gemcitabine plus nab-paclitaxel therapy as the first-line treatment for pancreatic cancer patients with distant metastases [15, 16]. For patients in whom these treatments are unsuitable owing to their systemic condition or age, gemcitabine monotherapy, S-1 monotherapy or gemcitabine plus erlotinib combination therapy is recommended. A phase III study conducted overseas demonstrated the survival benefits of the FOLFIRINOX regimen [37], combined gemcitabine plus nab-paclitaxel regimen [38], gemcitabine monotherapy [40], and the gemcitabine plus erlotinib regimen [41]. Thereafter, clinical trials were also conducted in Japan, and the efficacy and safety of these regimens were also confirmed in Japanese patients [42–45]. On the other hand, S-1 monotherapy has come to be recommended as a standard treatment on the basis of the results of a phase III study (GEST study) conducted in Japan and Taiwan [46, 47]. The GEST study examined the non-inferiority of S-1 monotherapy to gemcitabine monotherapy and the superiority of combined gemcitabine plus S-1 therapy over gemcitabine monotherapy, in terms of the survival outcomes. The non-inferiority of S-1 monotherapy was statistically confirmed (the median survival time was 8.8 months in the gemcitabine arm and 9.7 months in the S-1 arm; HR 0.96, *p* < 0.001). However, the superiority of the combined gemcitabine plus S-1 regimen could not be confirmed (the median survival time was 8.8 months in the gemcitabine arm and 10.1 months in the gemcitabine plus S-1 arm; HR 0.88, *p* = 0.15). Therefore, S-1 monotherapy has come to be regarded as a standard treatment in Asian countries [13, 15, 16]. However, S-1 therapy has been reported to have only marginal anti-tumor activity in Western populations [48], and is, therefore, not regarded as a valid treatment option in Western countries.

The second-line treatment regimens recommended in Japan are: (1) a fluorouracil-containing regimen after a first-line gemcitabine-containing regimen, (2) a gemcitabine-containing regimen after a first-line fluorouracil-containing regimen, and (3) pembrolizumab for microsatellite instability-high (MSI-H) [15, 16]. The fluorouracil-containing regimen consists of fluorouracil combined with folinate calcium and MM-398 (nanoliposomal irinotecan).

Tropomyosin receptor kinase (TRK) inhibitors have been reported to be useful for patients with solid tumors harboring neurotrophic receptor tyrosine kinase (NTRK) fusion genes. At the 2018 Annual Meeting of the European Society of Clinical Oncology (ESMO), the results from an integrated analysis of three studies (STARTRK-2 study, STARTRK-1 study and ALKA-372-001 study) conducted in patients with tumors harboring NTRK fusion genes were presented [49]. In these studies, entrectinib, a TRK inhibitor, was administered to 54 patients with soft-tissue sarcomas, non-small cell lung cancer, and others, and the response rate was 57.4%. Entrectinib was also approved in Japan in June 2019. Pancreatic cancer harboring NTRK fusion genes is reported to be extremely rare, accounting for less than 1% of all cases [50]. The Japanese guidelines are being revised to include entrectinib as a treatment option.

## Future of pancreatic cancer chemotherapy

### Immunotherapy

Programmed death-ligand 1 (PD-L1) (found on the surfaces of cancer cells and stromal cells) and programmed cell death protein 1 (PD-1) and cytotoxic T-lymphocyte-associated protein 4 (CTLA-4) (found on the surface of T cells) have been shown to play particularly important roles in the suppression of T-cell activation by cancer cells. Strong efficacy of such monoclonal antibodies against specific types of cancer that are known to show particularly high immunogenicity (e.g., malignant melanoma) has been reported. In 2011, ipilimumab (an anti-CTLA-4 antibody) was approved for the treatment of malignant melanoma in the US, followed by the approval and clinical introduction of two anti-PD-1 antibodies (nivolumab and pembrolizumab) and three anti-PD-L1 antibodies (atezolizumab, avelumab, and durvalumab) for the treatment of several types of cancer. Active efforts have also been made to develop similar therapies for pancreatic cancer. A phase II study of ipilimumab alone for unresectable pancreatic cancer was conducted, and the response rate was 0% (0 of 27 patients) [51]. Similarly, in a phase I study of BMS-936559 (an anti-PD-L1 antibody), the response rate was 0% (0 of 14 patients) [52]. In addition, a randomized phase II study was conducted to compare durvalumab (an anti-PD-L1 antibody) alone with combined durvalumab plus tremelimumab (an anti-CTLA-4 antibody) therapy [53]. The response rate and the median survival time were 0% (0 of 32 patients) and 3.6 months, respectively, in the monotherapy group, and 3% (1 of 32 patients) and 3.1 months, respectively, in the combined therapy group. Thus, the immune checkpoint inhibitors, even the two immune checkpoint inhibitors used in combination, failed to elicit any desirable treatment outcomes. Therefore, the development of immune checkpoint inhibitors for pancreatic cancer is currently focused on combination therapy with chemotherapeutic agents, based on the expectation of possible add-on effects to current standard treatments, such as combined gemcitabine plus nab-paclitaxel therapy and FOLFIRINOX therapy [54–56]. However, in regard to MSI-H pancreatic cancer, some studies of pembrolizumab monotherapy have shown encouraging results [57, 58]. MSI-H is considered as a target against which immune checkpoint inhibitors are effective.

One of the possible reasons why immune checkpoint inhibitors are less effective in patients with pancreatic cancer is that pancreatic cancer contains proliferating interstitial components with a few tumor-infiltrating T cells. Therefore, various studies have been conducted to develop treatments using immune checkpoint inhibitors in combination with drugs targeting the tumor microenvironment characterized by such proliferation of interstitial components and immune responses. Human tumor-infiltrating Treg cells, which suppress anti-tumor immunity, express high levels of the chemokine receptor CCR4. In a phase I study of nivolumab plus mogamulizumab, a monoclonal antibody targeting CCR4, for patients with solid tumors, the partial response, and stable disease rates in 15 patients with pancreatic cancer were 7% and 33%, respectively [59]. It is reported that pancreatic cancer is characterized by a high degree of infiltration by tumor-associated macrophages (TAMs) that inhibit anti-tumor T-cell activity, and that blocking colony-stimulating factor 1 receptor (CSF-1R) signaling—which supports the recruitment, differentiation, and maintenance of immunosuppressive macrophages in tumors—may lead to depletion of the TAMs and upregulation of T-cell checkpoints. In a phase 1a/b study, cabiralizumab, a monoclonal antibody targeting CSF-1R signaling, plus nivolumab four partial responses (13%) were observed in 31 patients with pancreatic cancer [60, 61]. At present, a randomized phase II study to compare the efficacy of nivolumab plus cabiralizumab with or without chemotherapy in patients with pancreatic cancer is in progress [62]. TGFβ is another main contributor to immune evasion and tumor progression. M7824 is a bifunctional fusion protein composed of two extracellular domains of TGF-βRII (a TGF-β “trap”) fused with a human IgG1 monoclonal antibody against PD-L1. In a phase I study of M7824 conducted in patients with advanced solid tumors, one and three of the five pancreatic cancer patients enrolled in the study showed partial response and stable disease, respectively [63]. Furthermore, several trials of immunotherapy-based treatment combinations with targeted agents are ongoing for patients with pancreatic cancer [64–66].

In Japan, clinical studies of immunotherapy using peptide vaccines are actively being conducted [67–69]. Among them, a randomized phase II study for the Wilms’ tumor gene 1 (WT1) vaccine showed promising results [69]; WT1, which is ranked as the top antigen among 75 tumor-associated antigens (TAAs) [70], is one of the most promising TAAs. In this study, gemcitabine plus WT1 vaccine tended to prolong the progression-free survival (HR 0.66; *p* = 0.084) and improve the OS (HR 0.82; *p* = 0.363) in comparison with gemcitabine monotherapy. Currently, a phase III study of a dendritic cell vaccine loaded with WT1 peptides (TLP0-001) is being conducted in Japan in patients with advanced pancreatic cancer refractory to standard chemotherapy [71–73]. Many clinical studies of immunotherapy using vaccines have also been conducted overseas. In particular, randomized-controlled trials of prime/boost vaccination with GVAX and CRS-207 have yielded encouraging results [74]. GVAX, which is composed of two irradiated, granulocyte–macrophage colony-stimulating factor (GM-CSF)-secreting allogeneic pancreatic cancer cell lines, administered 24 h after treatment with low-dose cyclophosphamide (Cy) to inhibit regulatory T cells, induced T-cell immunity against cancer antigens, including mesothelin. CRS-207, a live-attenuated *Listeria monocytogenes*—expressed mesothelin, induces innate and adaptive immunity. On the basis of preclinical synergy, a phase II randomized study was conducted to compare Cy/GVAX followed by CRS-207 (arm A) with Cy/GVAX alone (arm B) in patients with metastatic pancreatic cancer. A total of 90 patients were treated (arm A, *n* = 61; arm B, *n* = 29); the OS was 6.1 months in arm A versus 3.9 months in arm B (HR 0.59; *p* = 0.02). On the basis of the observed survival and favorable safety profile, Cy/GVAX and CRS-207 are being explored further as suitable treatments for pancreatic cancer.

CAR-T cells are engineered T cells from patients, which can recognize tumor antigens, by transfection of genes encoding B-cell epitopes and T-cell activation signals. CAR-T cells infused into patients elicit an immune response that specifically attacks only those cells, including cancer cells, which express the target proteins. Multinational phase II studies (ELIANA study, JULIET study) have demonstrated the efficacy of CAR-T-cell therapy in patients with diffuse large B-cell lymphoma (DLBCL) [75, 76]. Clinical studies of CAR-T-cell therapy for pancreatic cancer are also in progress, and the results are expected. A phase I study was conducted to evaluate the safety and efficacy of adoptive cell therapy with autologous mesothelin-specific CAR-T cells (CARTmeso cells) in six patients with chemotherapy-refractory metastatic pancreatic cancer [77]. The disease stabilized in two patients and the progression-free survival times were 3.8 and 5.4 months. In 18F-2-fluoro-2-deoxy-d-glucose (FDG)-positron emission tomography/computed tomography imaging performed to monitor the metabolic active volume (MAV) of individual tumor lesions, the total MAV remained stable in three patients and decreased by 69.2% in one patient with biopsy-proven mesothelin expression; in this patient, all liver lesions showed complete abrogation of FDG uptake at 1 month as compared to the baseline.

## Molecular-targeted therapy

KRAS, CDKN2A, TP53, and SMAD4 have been recognized as major driver genes in pancreatic carcinogenesis. Mutations in these genes are the most commonly encountered mutations in the majority of patients with pancreatic cancer, although potential therapeutic-target genes are limited to KRAS G12C and CDKN2A, which are found only in a small subgroup of patients. Thus, at present, there are no promising therapeutic agents for non-KRAS G12C, TP53, and SMAD4-mutated pancreatic cancer, which account for the majority of the cases. Therefore, selection of the treatment regimen based on gene mutations has yet to become standard strategies for patients with pancreatic cancer. Of the gene mutations, mutations of the DNA repair genes, such as BRCA1/2, PALB2, ATM, ATR, and ATRX, are the most common “highly actionable” alterations. It has been reported that in patients with gene mutations leading to homologous recombination deficiency (HRD), poly (ADP-ribose) polymerase (PARP) inhibitors exert anti-tumor effects by inducing cell death via synthetic lethality. In recent years, promising results have been reported from clinical studies of PARP inhibitors for patients with germline BRCA-mutated pancreatic cancer. According to the findings of the recently completed, international, phase-III POLO (Pancreas cancer OLaparib Ongoing) trial, treatment with the PARP inhibitor olaparib significantly reduced the risk of disease progression in patients with a germline BRCA1 or BRCA2 mutation and metastatic pancreatic cancer and disease that had not progressed during the first-line platinum-based chemotherapy [78]. In this study, 3315 patients with pancreatic cancer were screened for germline BRCA1/2 mutations, and 247 (7.5%) were found to have BRCA1/2 mutations. Of these 247 patients, 154 were randomized to receive olaparib or placebo. The primary endpoint, namely, progression-free survival, was significantly prolonged in the olaparib group as compared to the placebo group (median progression-free survival: 7.4 months vs. 3.8 months, HR 0.53, *p* = 0.004). Based on this result, the National Comprehensive Cancer Network (NCCN) guidelines in the United States includes the recommendation of olaparib as maintenance treatment for patients who have germline BRCA1/2 mutations, good PS, metastatic disease, and no disease progression after at least 4–6 month first-line chemotherapy [11]. Other PARP inhibitors such as veliparib and rucaparib are also being examined in clinical trials for pancreatic cancer [79–81].

Although *KRAS* mutations are predominant in pancreatic cancer, no effective therapeutic agent targeting *KRAS* mutations has been discovered until date. Recently, however, several candidate inhibitors of the KRAS G12C mutant protein have been reported. Among these, encouraging results were reported from a phase I study of AMG510, especially in a non-small cell lung cancer cohort [82]. On the other hand, pancreatic cancers with wild-type *KRAS*, although rare, account for only about 5% of all cases; *BRAF* and *EGFR* gene mutations and some fusion genes (*FGFR, ALK, NTRK, NRG1*, etc.) have also been reported to be detected at a relatively high frequency [83].

The “Know Your Tumor Project” initiative undertaken by the Pancreatic Cancer Patient Association (PanCAN) in the United States reported that 26% of patients with pancreatic cancer had actionable alterations, suggesting the possibility that therapy targeting these alterations could prolong the survival [84]. Recently, the NCCN guidelines have been updated to include the following: “Germline testing is recommended for any patient with confirmed pancreatic cancer using comprehensive gene panels for hereditary cancer syndromes” and “Tumor/somatic gene profiling is recommended for patients with locally advanced/metastatic disease who are candidates for anti-cancer therapy to identify uncommon mutations.” Thus, the guidelines recommend a search for actionable mutations, even if they are rare [11]. In the near future, guidelines in other countries may also follow these recommendations.

## Others

Metastatic pancreatic ductal adenocarcinoma is characterized by excessive hyaluronan (HA) accumulation in the tumor microenvironment, with elevation of the interstitial pressure and impaired perfusion. Preclinical studies have demonstrated that pegvorhyaluronidase alfa (PEGPH20) degrades HA, thereby increasing drug delivery. In a randomized phase II study, patients with previously untreated metastatic pancreatic ductal adenocarcinoma were randomly assigned to treatment with PEGPH20 plus nab-paclitaxel/gemcitabine (PAG) or nab-paclitaxel/gemcitabine (AG) [85]. The progression-free survival was significantly prolonged with PAG treatment in the overall subject population (HR 0.73; *p* = 0.049) and in patients with HA-high tumors (HR 0.51; *p* = 0.048). On the other hand, PEGPH20 combination with modified FOLFIRINOX caused increased toxicity and resulted in decreased treatment duration compared with modified FOLFIRINOX alone in another randomized study [86]. A global phase III study of PAG versus AG in patients with HA-high PDA is now ongoing [87].

Circulating tumor cells (CTCs) and circulating tumor DNA (ctDNA), as liquid biopsies, are an emerging minimally invasive tool with a unique potential for (a) determining the prognosis, (b) monitoring therapeutic responses and tumor recurrence in real time, (c) exploring therapeutic targets, and (d) potentially developing new drugs by studying metastatic cancer biology and drug resistance mechanisms [88–90]. In addition to CTCs and ctDNA, circulating tumor extracellular vesicles (e.g., exosomes), tumor-educated platelets (TEPs), and blood-based protein and metabolite markers also show early promise as biomarkers that could be used from cancer screening through to targeted treatments for pancreatic cancer. In particular, ctDNA is expected to be clinically useful in noninvasive molecular profiling for the novel actionable mutations [91]. Sequential real-time liquid biopsies could potentially allow early identification of resistance to cancer therapy in individual patients as an important hallmark of personalized cancer medicine. Detection and characterization of minimal residual disease after resection are another important aim and challenge for future studies [92].

## Conclusions

Steady progress has been made in the development of non-surgical therapies for advanced pancreatic cancer, and the prognosis of the patients is steadily improving, although the results are still far from satisfactory. Until now, the standard systemic treatment for pancreatic cancer has been limited to existing cytotoxic anti-cancer drugs. Recently, large-scale randomized-controlled studies of pre- and postoperative adjuvant therapies have been actively undertaken in an attempt to improve the prognosis of patients with resectable pancreatic cancer by introducing chemotherapies which have been commonly used for advanced pancreatic cancer, with or without radiotherapy, and this trend is expected to continue in the future. For some pancreatic cancers with actionable mutations, such as germline BRCA1/2 mutations, NTRK fusion mutation, and MSI-H, molecular-targeted therapy and immunotherapy are being introduced. It is expected that the mechanisms of initiation and development of pancreatic cancer will be better elucidated and that targeted treatments that would yield marked tumor shrinkage and survival prolongation will be developed. In addition to developing highly effective and safe anti-cancer treatments, it is also important to ensure that supportive and palliative care is available for efficient implementation of such anti-cancer treatments, and multidisciplinary cooperation and collaboration with the community should be promoted, because both improvement of the patient prognosis and improvement of the quality of life are major goals of treatment of this disease.
